# PD-L1 and BAP1 as Prognostic Biomarkers in Malignant Pleural Mesothelioma

**DOI:** 10.3390/cells15020183

**Published:** 2026-01-19

**Authors:** Milija Gajić, Vesna Ćeriman Krstić, Natalija Samardžić, Ivan Soldatović, Sofija Glumac, Milena Jovanović, Milan Savić, Mihailo Stjepanović, Spasoje Popević, Ruža Stević, Nikola Čolić, Katarina Lukić, Vladimir Milenković, Ivan Milivojević, Ivana Sekulović Radovanović, Dragana Jovanović

**Affiliations:** 1 Clinic for Pulmonology, University Clinical Center of Serbia, 11000 Belgrade, Serbia; 2 Faculty of Medicine, University of Belgrade, 11000 Belgrade, Serbiadrcola12@gmail.com (N.Č.);; 3 Institute of Medical Statistics, Faculty of Medicine, University of Belgrade, 11000 Belgrade, Serbia; 4 Department of Pathology, Faculty of Medicine, University of Belgrade, 11000 Belgrade, Serbia; 5 Clinic for Thoracis Surgery, University of Belgrade, 11000 Belgrade, Serbia; 6 Center for Radiology, University of Belgrade, 11000 Belgrade, Serbia; 7 Internal Medicine Clinic “Akta Medica”, 11000 Belgrade, Serbia

**Keywords:** malignant pleural mesothelioma, prognostic biomarker, PD-L1, BAP1

## Abstract

Malignant pleural mesothelioma (MPM) is a very aggressive tumor. The prognostic value of PD-L1 and BAP1 expression has been investigated in many studies. A retrospective study was conducted that analyzed PD-L1 and BAP1 expression as prognostic biomarkers in patients with MPM. The study included 53 patients with MPM. PD-L1 expression ≥ 1% was found in 39.6%, and BAP1 loss was found in 81.1% of patients. The median overall survival (mOS) was 11 months. Subtype of MPM (*p* = 0.045), early tumor stage (*p* = 0.049), therapy (*p* = 0.002), and good PS (0–1) (*p* = 0.012) were associated with better survival. Expression of PD-L1 and BAP1 did not show statistical significance regarding OS, but OS was numerically shorter in patients with PD-L1 ≥ 10% (5 vs. 12 months) and longer in patients with BAP1 loss (12 vs. 4 months). In patients with PD-L1 ≥ 1% and BAP1 loss, the median progression-free survival (mPFS) was numerically longer (10 vs. 7 months) but in patients with PD-L1 ≥ 1% and BAP1 positivity, PFS was statistically significantly shorter (1 vs. 7 months, *p* = 0.048). Our results did not show that PD-L1 and BAP1 are prognostic biomarkers for MPM, but positive PD-L1 expression and BAP1 loss were associated with worse survival in patients with MPM.

## 1. Introduction

Malignant pleural mesothelioma is a rare and very aggressive tumor of mesothelial cells that, in the majority of cases, affects the pleura and peritoneum, while the pericardium and the tunica vaginalis of the testis are affected less often [1,2,3]. Globally, about 30,000 new cases are registered annually [3,4]. The largest number of cases of malignant pleural mesothelioma is associated with exposure to asbestos mineral fibers, while a smaller number of causes include non-asbestos mineral fibers (erionite and fluoro-edenite), ionizing radiation, chronic inflammation, SV40 virus, and inactivating mutations in tumor suppressor genes (BAP1, NF2, TP53, and SETD2) [1,3,5,6]. The median age of the patients at the time of diagnosis is 70 years [7]. The median overall survival (mOS) with the multimodality therapy (MMT) approach is 20–29 months [1]. Different clinical trials (CheckMate 743, MAPS) showed that the 3-year OS rate was 23% for patients with unresectable pleural mesothelioma treated with dual immunotherapy compared to 15% of patients treated with chemotherapy [8,9]. Postoperative radiotherapy reduces the rate of local disease recurrence but has no influence on overall survival [7,10]. Programmed cell death 1 (PD-1) receptor is found on the surface of T-lymphocytes, B-lymphocytes, and NK cells. Tumor cells can express programmed death-ligand 1 (PD-L1), by which they bind to the PD-1 receptor on T-lymphocytes and thus suppress the T-cell immune response, which results in a decrease in the activation of proliferating T-lymphocytes, the release of cytokines, and the cytolytic activity of PD-1 positive lymphocytes [11]. The previous results indicate that PD-L1 expression was found in 40% of mesothelioma cases, that the frequency of PD-L1 expression in sarcomatoid mesothelioma is significant, and that it is associated with a worse prognosis [12,13,14]. The epithelioid type of mesothelioma shows a lower level of PD-L1 positivity [15]. BRCA1-associated protein-1 (BAP1) is localized on the third chromosome (3p21). Loss of BAP1 is a hallmark malignant transformation. BAP1 mutation is present in 60% of malignant pleural mesothelioma [16]. It is more frequent in the epithelioid histological type [16,17]. Observational studies confirmed longer survival in patients with epithelioid subtype of mesothelioma and BAP1 alteration [10,18]. BAP1 tumor predisposition syndrome (BAP1 TPS) is an autosomal dominant disease in which there is a heterogeneous mutation of BAP1 and represents a high risk for the occurrence of mesothelioma and other tumors (melanoma) [19,20]. The European Organization for Research and Treatment of Cancer (EORTC) and Cancer and Leukemia Group B (CALGB) suggested poor performance status, non-epithelioid histological type of mesothelioma, male sex, low hemoglobin level, thrombocytosis, leukocytosis, and elevated lactate dehydrogenase values as predictors of poor clinical response to the applied therapy [21,22]. The prognostic value of PD-L1 and BAP1 expression has been investigated in different clinical studies, but the results are controversial, unlike the demonstrated negative correlation of high levels of PD-L1 expression in kidney and stomach cancer and positive correlation in colorectal and thymus cancer [23].

Since it was already known that patients with BAP1 loss had better OS if they were treated with chemotherapy, we wanted to investigate whether PD-L1 as a biomarker has an impact on outcomes in patients with BAP1 loss and BAP1 positivity.

## 2. Materials and Methods

### 2.1. Study Population

The current retrospective study was conducted in the Clinic for Pulmonology, University Clinical Center of Serbia. The study included 89 patients with MPM, of whom 53 had available tumor tissue for further analysis. Patients who were included were older than 18 years, and they were diagnosed with malignant pleural mesothelioma between 2008 and 2018. The patients who were included were all treatment naïve. The stage of the disease was determined according to the IMIG TNM staging system (7th edition) [24]. Clinical data were obtained from medical history and included age, gender, histological type (according to the EURACAN/IASLC histological classification [15]), stage, performance status (PS), treatment, and survival outcomes. The aim of the study was to analyze the clinical characteristics of patients with MPM, the expression of PD-L1 and BAP1, and their correlation with clinical outcomes. In order to determine whether PD-L1 could be a prognostic factor for survival, we used a cut-off point of ≥1%. Patients with PD-L1 ≥ 1% were further categorized as PD-L1 “low” (<10%) and “high” (≥10%) [23]. BAP1 expression was determined by immunohistochemical analysis, and the result was presented as positive (present expression) and negative (loss of BAP1).

### 2.2. Tissue Samples

The tissue samples of 53 patients with MPM were obtained through diagnostic procedures (pleural biopsy, surgery). All formalin-fixed paraffin-embedded samples (FFPE) were processed and stored at room temperature. PD-L1 expression was determined using Clone 22C3 pharmaDX (Dako Omnis, Agilent, SC, USA, flex ready to use). PD-L1 tumor proportion score (TPS) was calculated as a percentage of at least 100 tumor cells with complete or partial membrane staining [21]. Positive PD-L1 samples were defined by a score of TPS ≥ 1%. Clone C-4 (Santa Cruz, Dallas, TX, USA, sc-28383) was used to determine BAP1 expression.

### 2.3. Treatment

Patients were treated according to the current National Comprehensive Cancer Network (NCCN) guidelines, using surgical procedures (pleurectomy/decortication (P/D) or extrapleural pneumonectomy (EPP)), platinum-based chemotherapy (CHT), radiotherapy (RT), or best supportive care (BSC). Multimodality therapy (MMT) is defined by the use of surgery and chemotherapy with or without the use of radiotherapy. Our group of patients was not treated with immunotherapy or targeted agents.

### 2.4. Statistical Analyses

All data were analyzed using SPSS 29.0 (IBM Corp. Released 2023. IBM SPSS Statistics for Windows, Version 20.0. Armonk, NY, USA: IBM Corp.) and R 3.4.2 (R. Core Team (2017). R: A language and environment for statistical computing. R Foundation for Statistical Computing, Vienna, Austria. URL https://www.R-project.org/). Descriptive and analytical statistical methods were used in this study. Of the descriptive methods, absolute and relative numbers (N, %), measures of central tendency (arithmetic mean, median), and measures of dispersion (standard deviation, interquartile range) were used. Analytical methods will be used, including difference tests, the parametric *t*-test, the non-parametric χ2 test, and the Mann–Whitney test. The Kaplan–Meier curve with a confidence interval (CI) of 95%, a log-rank test with a significance level of 5% (chi-square *p* = 0.05), and a Cox regression analysis will be used for survival analysis. A two-sided *p*-value < 0.05 was considered statistically significant.

## 3. Results

### 3.1. Study Population and Clinical Characteristics

The study included 53 patients for whom tumor tissue samples were available for further analysis. The mean age of the study population was 65 years (range, 46–82) and 69.8% were male. Epithelioid was the most common histological subtype, present in 41 patients (77.4%). The majority of patients, 37 (69.8%), were in the early stage (I and II). Chemotherapy was applied in 32 patients (60.4%), while 15.1% (*n* = 8) of patients received multimodality treatment. Moreover, 86.8% (n = 46) of patients had an Eastern Cooperative Oncology Group (ECOG) Performance Status (PS) of 0–1. The PD-L1 TPS ≥ 1% was found in 39.6% patients and 18.9% of patients had PD-L1 ≥ 10%. A loss of BAP1 was found in 81.1% of patients. The patients’ characteristics and expression of PD-L1 and BAP1 are presented in Table 1.

### 3.2. Associations of Clinical Characteristics with Expression of PD-L1 and BAP1

Clinical characteristics, including gender, age, stage, treatment, and PS, had no significant association with PD-L1 expression, while a significant association was found with epitheloid subtype and PD-L1 < 1% (*p* = 0.033). A significant association was found between PD-L1 < 10%, stage, and treatment; further, PD-L1 < 10% was more frequent in early stages (*p* = 0.050), and also the majority of the patients with PD-L1 < 10% received chemotherapy (*p* = 0.041). BAP1 loss was associated with the early stage (*p* = 0.05). Associations of clinical characteristics with expression of PD-L1 and BAP1 are presented in Table 2.

### 3.3. Associations of Clinical Characteristics with PFS

Median progression-free survival (mPFS) was seven months. Patients with sarcomatoid histological subtype (sarcomatoid vs. biphasic, 1 vs. 13.5 months, *p* = 0.008), later stage (early vs. late, 9 vs. 4 months, *p* = 0.034), and PS 2–3 (0–1 vs. 2–3, 7.5 vs. 1 months, *p* = 0.042) had significantly shorter median PFS. PD-L1 expression and BAP1 loss had no significant influence on mPFS, but mPFS was shorter in patients with PD-L1 expression ≥ 10% (7 vs. 3 months, *p* = 0.100) and longer in patients with BAP1 loss (9 vs. 4 months, *p* = 0.264). A univariate survival analysis for PFS adjusted for clinical variables is presented in Table 3.

### 3.4. Survival Analysis

The mOS was 11 months. Histological subtype had a significant influence on survival (*p* = 0.045). Patients with the biphasic histological subtype had the longest OS (29.5 months) numerically, and those with the sarcomatoid subtype had the shortest OS (1 month). The disease stage had a significant influence on the OS; thus, patients with the early stage of the disease had a significantly longer OS compared to the patients with the late stage (12.5 vs. 9 months, *p* = 0.045). Patients treated with MMT had the longest OS (26.5 months) compared to patients treated with other applied treatments (CHT, OP) (11 and 12 months, respectively) or BSC (6 months) (*p* = 0.002). Patients with PS 0–1 had a significantly longer OS compared to patients with PS 2–3 (12.5 vs. 1 months, *p* = 0.012). The expression of PD-L1 ≥ 1% did not have a significant influence on the OS compared to the OS of patients with PD-L1 < 1% (12 vs. 11 months, *p* = 0.799). A univariate survival analysis for OS adjusted for clinical variables is presented in Table 4.

**Figure 1:**
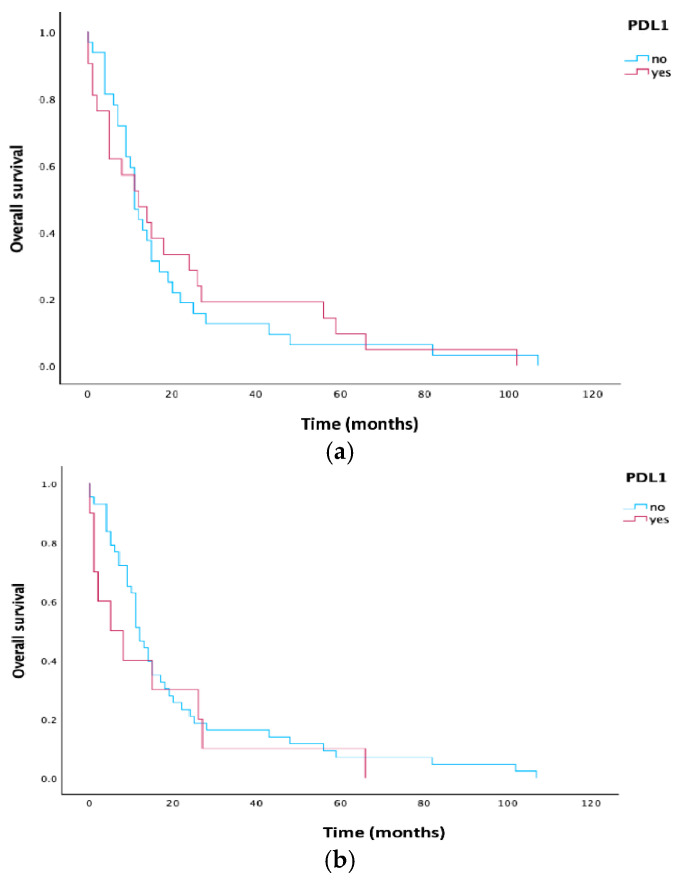
(**a**) Kaplan–Meier curves estimate for OS according to PD-L1 expression in all patients: red line for patients with PD-L1 expression ≥ 1%, blue line for patients with PD-L1 expression < 1%. (**b**) Kaplan–Meier estimates for OS according to PD-L1 expression in all patients: red line for patients with PD-L1 expression ≥ 10%, blue line for patients with PD-L1 expression < 10%. Abbreviation: OS, overall survival; PD-L1, programmed cell death ligand 1.

In the subgroup of patients with PD-L1 ≥ 10%, OS was shorter compared to patients with PD-L1 < 10% (5 vs. 12 months, *p* = 0.464). Kaplan–Meier curves for PD-L1 are illustrated in Figure 1.

**Figure 2:**
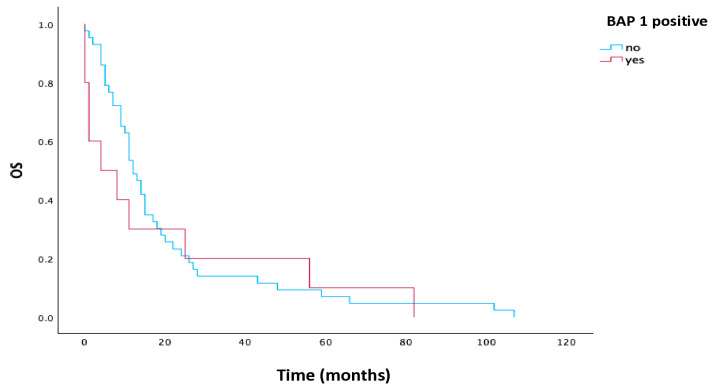
Kaplan–Meier estimates for OS according to BAP1 in all patients. Abbreviations: OS, overall survival; BAP1, BRCA1-associated protein-1.

The BAP1 loss had no significant influence on median OS (*p* = 0.541), but median OS was longer in patients with BAP1 loss (12 vs. 4 months). Kaplan–Meier curves for BAP1 are illustrated in Figure 2.

**Figure 3:**
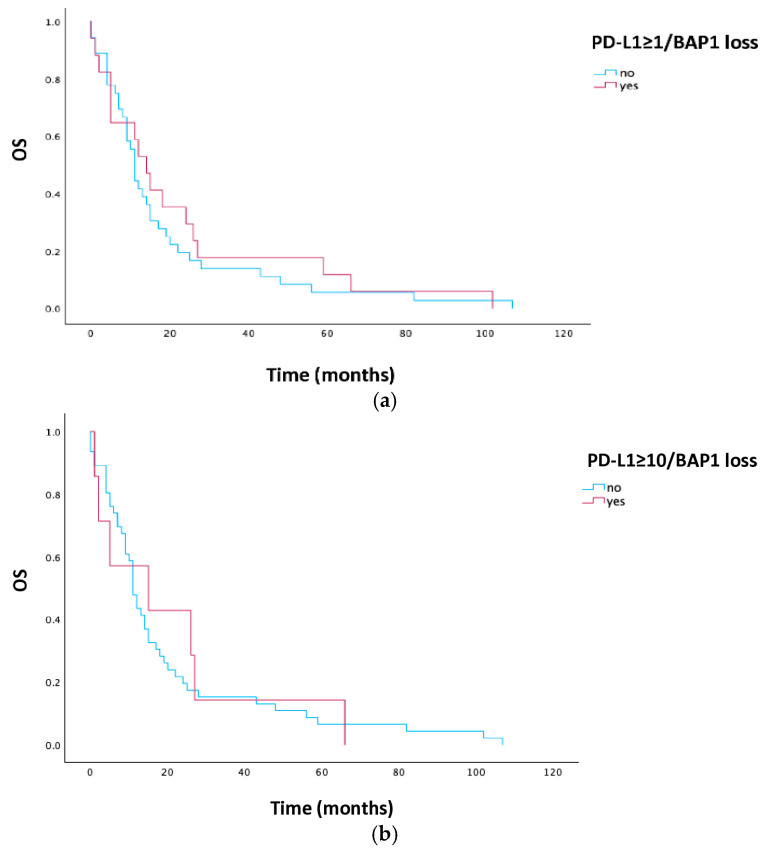
Kaplan–Meier estimates for OS according to PD-L1 and BAP1 in all patients. (**a**) OS in patients with PD-L1≥1% and BAP1 loss; (**b**) OS in patients with PD-L1≥10% and BAP1 loss. Abbreviations: OS, overall survival; PD-L1, programmed cell death ligand 1; BAP1, BRCA1-associated protein-1.

Patients with PD-L1 ≥ 1% and BAP1 loss did not have a statistically significant longer median OS (14 vs. 11 months, *p* = 0.541), nor did those in the subgroup of PD-L1 ≥ 10% and BAP1 loss (15 vs. 11 months, *p* = 0.820). Kaplan–Meier curves for PD-L1 and BAP1 are illustrated in Figure 3.

**Figure 4:**
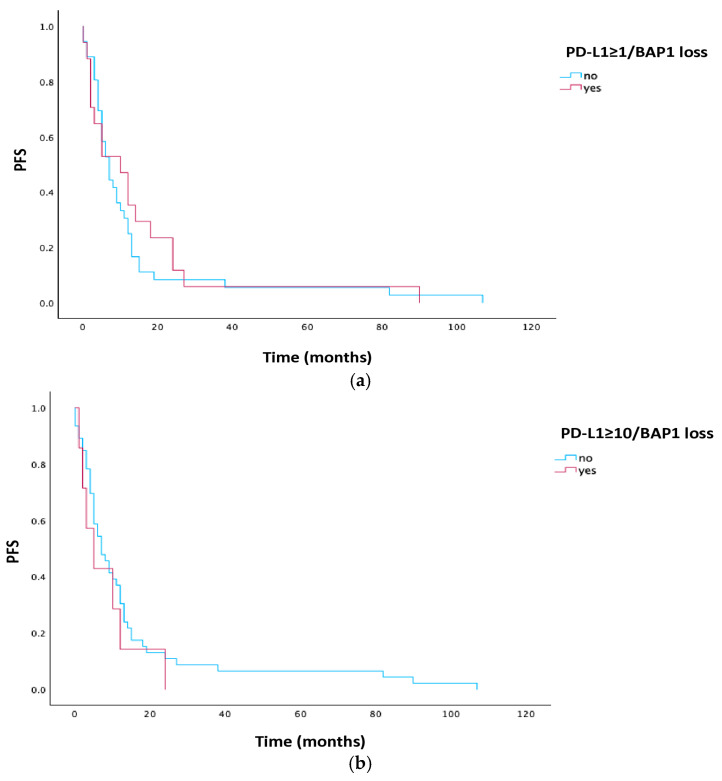
(**a**). Kaplan–Meier estimates for PFS in patients with PD-L1 ≥ 1 and BAP1 loss. (**b**). Kaplan–Meier estimates for PFS in patients with PD-L1 ≥ 10 and BAP1 loss. Abbreviations: PFS, progression-free survival; PD-L1, programmed cell death ligand 1; BAP1, BRCA1-associated protein-1.

The PFS in patients with PD-L1 ≥ 1% and BAP1 loss was numerically longer, but without statistical significance (10 vs. 7 months, *p* = 0.589), while in the PD-L1 ≥ 10% group and BAP1 loss expression, PFS was numerically shorter (5 vs. 7 months, *p* = 0.443). Kaplan–Meier curves for PD-L1 and BAP1 are illustrated in Figure 4.

**Figure 5:**
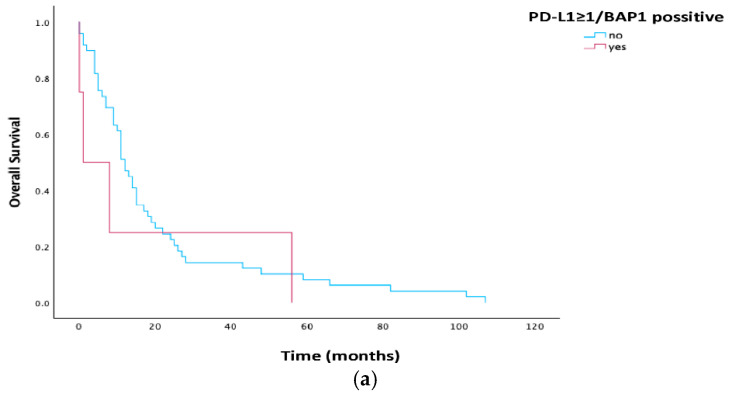

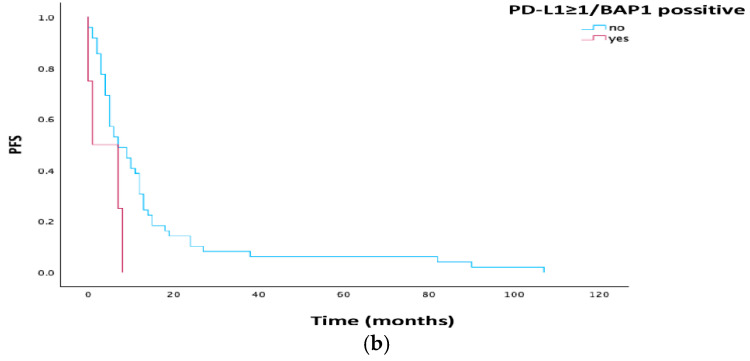
(**a**,**b**). Kaplan–Meier estimates for OS and PFS according to PD-L1 ≥ 1% and BAP1 positivity in all patients. Abbreviations: PFS, progression-free survival; PD-L1, programmed cell death ligand 1; BAP1, BRCA1-associated protein-1.

Patients with PD-L1 ≥ 1% expression and BAP1 positivity had statistically significant shorter median PFS (1 vs. 7 months, *p* = 0.048), while the impact on median OS had no statistical significance (*p* = 0.457). Kaplan–Meier curves for PD-L1 and BAP1 positivity is illustrated in Figure 5.

**Figure 6:**
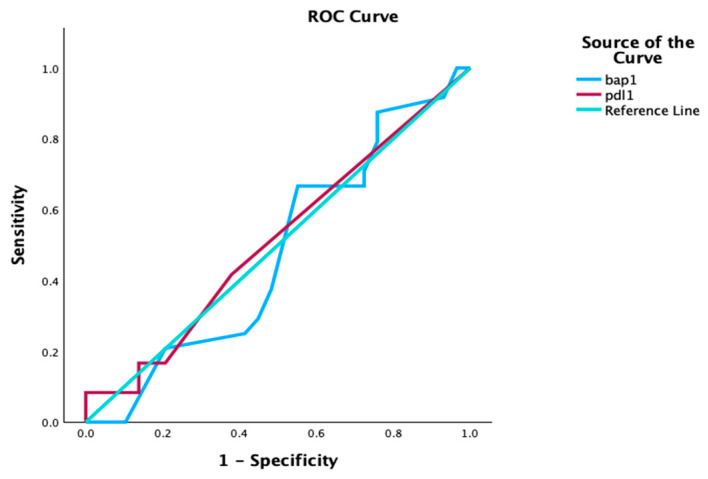
ROC estimates for OS according to PD-L1 and BAP1 in all patients. OS, overall survival; PD-L1, programmed cell death ligand 1; BAP1, BRCA1-associated protein-1.

The expressions of PD-L1 and BAP1 are not indicators of one-year survival (*p* = 0.872; *p* = 0.782). ROC curves for PD-L1 and BAP1 are illustrated in Figure 6.

## 4. Discussion

Malignant pleural mesothelioma is an aggressive tumor that is most often associated with exposure to asbestosis with an OS of 10 months and a 5-year survival rate of 5% (19, 25, 26). Since 2003, pemetrexed in combination with platinum has been the standard of care for unresectable MPM with a median OS of 12.7 months and median PFS of 7.7 months [4].

The clinical characteristics and expression of PD-L1 and BAP1 as prognostic biomarkers in patients with confirmed MPM were analyzed. The median age at the time of diagnosis was 65 years, with a predominance of male gender (69.8%) and of the epithelioid subtype (77.4%). The majority of patients had the early stage of disease (I and II) (69.8%), received CHT based on Pemetrexed/Platinum (60.4%), and had ECOG PS 0–1 (86.8%). The clinicopathological characteristics of our patients are not different from other studies except for the disease stage [21,22,23,25,26]. The median OS of patients in our study was 11 months, in contrast to 6 months in the study conducted by Pezzuto et al. [27] and 9 months in the study conducted by Ghanim et al. [28]. The majority of patients included in those studies were in the late stage of disease, and in our study the majority of patients were in the early stage of disease. Cedres et al. [29] reported a median OS of 21.3 months, which was significantly longer compared to the one in our study, and it could be explained by the good PS of the patients, the predominance of epithelioid histological subtype, and the fact that 10% of patients were included in clinical trials in a first-line setting. Also, approximately 15% of patients were treated with immunotherapy in second and further lines.

The clinicopathological characteristics of patients that had a significant association with survival were as follows: histological type, stage, treatment, and PS. Patients with the biphasic histological type had the longest median OS (25 months), in contrast to epithelioid and sarcomatoid (12 and 1 months, respectively). Our results are in contrast with other studies in which patients with epithelioid-type MPM had better survival [23,27,29]. The possible explanation could be the fact that our patients with the biphasic histological subtype were all in the early stage of disease, had good PS, and were treated with multimodality treatment.

Patients with the early stage had a significantly longer OS (12 vs. 9.5 months, *p* = 0.049), as did patients who were treated with MMT compared to other types of treatment (26 months, *p* = 0.002), which was also shown in the study of Brcic et al. [23]. The median OS of patients with ECOG PS 0–1 was 12.5 vs. 1 months in patients with PS 2–3 (*p* = 0.012). We did not find other studies that included patients with ECOG PS 2–3.

The median PFS in our study was seven months. It is not different from the results of other studies [21]. The clinicopathological characteristics of the patient that significantly influenced median PFS were as follows: histological type (biphasic vs. epithelioid and sarcomatoid, 13.5 vs. 7 and 1 months, *p* = 0.008), early stage (9 vs. 4 months, *p* = 0.034), and PS 0–1 (7.5 vs. 1 months, *p* = 0.042). Our results showed that stage, PS, and histology could be the prognostic factors.

The relationship between PD-L1 expression and clinicopathological characteristics, OS, and PFS has been investigated in many studies, but the prognostic significance is still controversial. In our study, 39.4% patients were PD-L1 positive (≥1%) and 60.4% of patients were PD-L1 negative (<1%). An association between PD-L1 expression and clinicopathological parameters (histological subtype, stage, therapeutic modality) was observed. Patients with PD-L1 < 1% had a more frequent epithelioid histological type (*p* = 0.033), while other clinicopathological parameters were not significantly associated with PD-L1. Illini et al. [22] reported a significant association between PD-L1 TPS ≥ 1% and histological subtype (epithelioid vs. non-epithelioid, *p* = 0.045, *p* = 0.024) and concluded that the non-epithelioid subtype was more common in patients with PD-L1 ≥ 1%. Some previous studies reported the significant association between PD-L1 ≥ 1% and the non-epithelioid histological subtype [11,30,31,32]. In contrast to previous studies, Brcic et al. [23] reported no significant association between PD-L1 and histological subtype and explained it by different cut-off values (PD-L1 “high” was defined as ≥ 10%) as well as the relatively low ratio of patients with non-epithelioid MPM. The association between stage and treatment modality was not found in the patients with PD-L1 < 1% (*p* = 0.686, *p* = 0.551), but in the group of patients with PD-L1 < 10%, the majority of patients were in the early stage (*p* = 0.05) and received chemotherapy (*p* = 0.041). Illini et al. [22] reported a significant association between PD-L1 ≥ 1% and stage (early vs. late, *p* = 0.034, *p* = 0.036) and concluded that a higher expression of PD-L1 was present in the later stages. In other studies, no statistically significant association between PD-L1 expression and stage was found [23].

Response to CHT (complete response, partial response, stable disease, progression disease) in our study was not significantly associated with PD-L1 expression, even in the subgroup analysis (*p* = 0.577, *p* = 0.238). Other studies did not report an association between response to treatment and PD-L1 expression.

In our study, median OS in patients with PD-L1 ≥ 1% was 12 months, while in patients with PD-L1 < 1% it was 11 months, which was similar to previous studies (15.3 vs. 20.0 months) [22]. There was no correlation between PD-L1 and OS, even in subgroups (low vs. high), but it was observed that patients with PD-L1 < 1% had numerically longer survival (12 vs. 5 months, *p* = 0.464). In contrast to the study of Brcic et al. [23], the “low” subgroup (<10%) had better OS (*p* < 0.001, HR 0.39), thus the PD-L1 expression was suggested as a significant prognostic factor for OS. Some studies have shown a statistically significant lower risk of death (*p* = 0.007, HR 0.16) in PD-L1-positive patients compared to PD-L1-negative patients [26]. A study by Dursun et al. [25] showed no statistically significant difference in survival between PD-L1-positive and PD-L1-negative patients (*p* > 0.05).

In our study, median PFS was seven months, and there was no statistically significant difference in patients with positive and negative PD-L1 expression (*p* = 0.860, *p* = 0.100). The statistically significant association between PD-L1 and median PFS was also shown in the study by Aviles-Salas et al. [21] where median PFS was 8.7 months, and 4.9 months in the PD-L1 ≥ 1% group compared to 10.8 months in the PD-L1 < 1% group (*p* = 0.008), while in the high PD-L1 subgroups it was 4.96 months compared to 10.8 months in the low PD-L1 group (*p* = 0.002). Brosseau et al. [26] reported a shorter median PFS in patients with PD-L1 expression ≥ 1% (6.9 months) compared to PD-L1 < 1% (9.5 months) but with no statistically significant difference. A statistically significant difference was observed in subgroups with PD-L1 expression > 50% and 1–50% (6.2 vs. 9.2 months, *p* = 0.001).

One previous study showed that BAP1 loss was present in MPM, and it may be associated with poor prognosis (*p* < 0.05) [30]. The BAP1 loss is very specific (81–99%) in MPM, but the sensitivity is 30–67% [33], which corresponds to the results of our study, where BAP1 loss was registered in 81.1% of patients. In previous studies, no significant association with clinicopathological characteristics was found, except for epithelioid histological subtype [16,22,33,34]. In our study, clinicopathological characteristics (age, sex, therapy modality, PS) were not significantly associated with BAP1 loss, except for stage, where there was a significant association with BAP1 loss in earlier stages (*p* = 0.050). In our study, patients with BAP1 loss had a longer median OS compared to patients with BAP1 positivity (12 vs. 4 months), but there was no statistical difference between the studied groups (*p* = 0.541). Similar results were shown in the study by Illini et al. [22], where longer survival was observed in patients with BAP1 loss (20 vs. 11.3 months, *p* = 0.153). Different results were shown in a study in which 60 patients with MPM were analyzed, and the 82% of patients that were BAP1-negative had a median OS of 14.8 months, which contrasted that of the BAP1-positive patients, whose OS was 18.1 months. However, multivariate analysis did not show significant differences between groups [35]. Retrospective studies also showed longer survival in patients with BAP1 loss but not PFS [35]. The results of our study showed a numerically longer time to disease progression in patients with BAP1 loss (9 vs. 4 months, *p* = 0.264), but without statistical significance.

The association between BAP1 loss, PD-L1 expression, and survival has been investigated. The results of our study showed no significant impact of BAP1 loss and expression of PD-L1 ≥ 1% on patient survival (*p* = 0.541) or on time to disease progression (*p* = 0.589). In previous studies, patients with PD-L1 ≥ 1% and BAP1 positivity had worse survival (*p* = 0.045, *p* = 0.059), which was also shown in our patients (1 vs. 12 months, *p* = 0.457), while mPFS was significantly shorter in patients with PD-L1 ≥ 1% and BAP1 positivity (*p* = 0.048) [22].

The presented study has some limitations. It was a retrospective study with a relatively small number of patients, and a small number of biopsy samples were used for PD-L1 and BAP1 testing in the majority of cases. Since small biopsies were used, the results of testing should be interpreted with caution. Also, the majority of cases were of the epithelioid histological subtype, and thus, the results cannot be generalized to the whole population of MPM. Further, our patients were not treated with immunotherapy, and new guidelines recommend immunotherapy as the first therapeutic option. Considering the above mentioned limitations, it is necessary to conduct a larger study with a larger number of patients.

## 5. Conclusions

In conclusion, histological subtype, stage, PS, and MMT could be prognostic factors for OS and PFS. PD-L1 and BAP1 are not indicators of one-year survival, but PD-L1 expression and BAP1 loss are associated with worse survival. The results of our study and previous studies showed the necessity of discovering new prognostic factors.

## Figures and Tables

**Table 1:** Patients’ characteristics, PD-L1 and BAP1 expression.

	N	%
Age		
≥65	32	60.4
<65	21	39.6
Sex		
Male	37	69.8
Female	16	30.2
Histology		
Epitheloid	41	77.4
Bifasic	2	3.8
MM	4	7.5
Sarcomatoides	6	11.3
Stage		
Early (I/II)	37	69.8
Late (III/IV)	16	30.2
Treatment		
CHT	32	60.4
Surgery	1	1.9
MMT	8	15.1
BSC	12	22.6
PS		
0–1	46	86.8
2–3	7	13.2
PD-L1		
TPS < 1%	32	60.4
TPS ≥ 1%	21	39.6
TPS < 10%	43	81.1
TPS ≥ 10%	10	18.9
BAP1		
Loss	43	81.1
Positive	10	18.9

Abbreviations: MM, malignant mesothelioma histologically nonspecific; MMT, multimodality treatment; CHT, chemotherapy; BSC, best supportive care; PD-L1, programmed cell death ligand 1; TPS, tumor proportion score; BAP1, BRCA1-associated protein-1.

**Table 2:** Associations of clinical characteristics with expression of PD-L1 and BAP1.

	PD-L1		PD-L1		BAP1	
	<1%	≥1%	<10%	≥10%	Loss	Positive
Age (N, %)						
≥65	17 (53.1)	15 (71.4)	27 (62.8)	5 (50.0)	26 (60.5)	4 (40.0)
<65	15 (46.9)	6 (28.6)	16 (37.2)	5 (50.0)	17 (39.5)	6 (60.0)
	*p* = 0.183		*p* = 0.492		*p* = 1.000	
Sex (N, %)						
Male	22 (68.8)	15 (71.4)	29 (67.4)	8 (80.29)	29 (67.4)	8 (80.0)
Female	10 (31.3)	6 (28.6)	14 (32.6)	2 (20.2)	14 (32.6)	2 (20.0)
	*p* = 0.835		*p* = 0.704		*p* = 0.704	
Histology (N, %)						
Epitheloid	26 (81.3)	15 (71.4)	35 (81.4)	6 (60.0)	34 (79.1)	7 (70.0)
Bifasic	1 (3.1)	1 (4.8)	1 (2.3)	1 (10.0)	2 (4.7)	0 (0.0)
MM	4 (12.5)	0 (0.0)	4 (9.3)	0 (0.0)	4 (9.3)	0 (0.0)
Sarcomatoides	1 (3.1)	5 (23.8)	3 (7.0)	3 (30.3)	3 (7.0)	3 (30.0)
	***p* = 0.033**		*p* = 0.087		*p* = 0.180	
Stage (N, %)						
Early (I/II)	23 (71.9)	14 (66.7)	33 (76.7)	4 (40.0)	33 (76.7)	4 (40.0)
Late (III/IV)	9 (28.1)	7 (33.3)	10 (23.3)	6 (60.0)	10 (23.3)	6 (60.0)
	*p* = 0.686		***p* = 0.050**		***p* = 0.050**	
Treatment (N, %)						
CHT	21 (65.6)	11 (52.4)	29 (67.4)	3 (30.0)	25 (58.1)	7 (70.0)
Surgery	0 (0.0)	1 (4.89)	1 (2.3)	0 (0.0)	1 (2.3)	0 (0.0)
MMT	4 (12.5)	4 (19.0)	4 (9.3)	4 (40.0)	8 (18.6)	0 (0.0)
BSC	7 (21.9)	5 (23.8)	9 (20.9)	3 (30.0)	9 (20.9)	3 (30.0)
	*p* = 0.551		***p* = 0.041**		*p* = 0.489	
PS (N, %)						
0–1	29 (90.6)	17 (81.0)	29 (90.6)	17 (81.0)	39 (90.7)	7 (70.0)
2–3	3 (9.4)	4 (19.0)	3 (9.4)	4 (19.0)	4 (9.3)	3 (30.0)
	*p* = 0.415		*p* = 0.415		*p* = 0.114	

Abbreviations: MM, malignant mesothelioma histologically nonspecific; MMT, multimodality treatment; CHT, chemotherapy; BSC, best supportive care; PD-L1, programmed cell death ligand 1; BAP1, BRCA1-associated protein-1.

**Table 3:** Univariate survival analysis for PFS adjusted for clinical variables.

	PFS		95.0% CI		*p* Value
	N	Median	Lower	Upper	
Age					0.477
≥65	32	7.0	5.0	13.0	
<65	21	7.5	5.0	12.0	
Sex					0.831
Male	37	7.0	5.0	12.0	
Female	16	7.0	5.0	13.0	
Histology					**0.008**
Epitheloid	41	7.0	5.0	12.0	
Bifasic	2	13.5	12.0	15.0	
MM	4	10.5	7.0	13.0	
Sarcomatoides	6	1.0	0.0	7.0	
Stage					**0.034**
Early (I/II)	37	9.0	5.0	12.0	
Late (III/IV)	16	4.0	2.0	13.0	
Treatment					0.345
CHT	32	6.0	5.0	11.0	
Surgery	1	12.0	0.0	0.0	
MMT	8	13.5	3.0	24.0	
BSC	12	6.0	1.0	13.0	
PS					**0.042**
0–1	46	7.5	5.0	12.0	
2–3	7	1.0	0.0	90.0	
PD-L1					
TPS < 1%	32	7.0	3.311	10.689	0.860
TPS ≥ 1%	21	7.0	1.393	12.607	
TPS < 10%	43	7.0	3.145	10.855	0.100
TPS ≥ 10%	10	3.0	0.000	7.649	
BAP1					
Loss	43	9.0	4.726	13.274	0.264
Positive	10	4.0	0.0	10.198	

Abbreviations: PFS, progression-free survival; MM, malignant mesothelioma histologically nonspecific; MMT, multimodality treatment; CHT, chemotherapy; BSC, best supportive care; PD-L1, programmed cell death ligand 1; TPS, tumor proportion score; BAP1, BRCA1-associated protein-1.

**Table 4:** Univariate survival analysis for OS adjusted for clinical variables.

	OS		95.0% CI		*p* Value
	N	Median	Lower	Upper	
Age					
≥65	32	11.0	6.0	17.0	0.856
<65	21	11.0	11.0	19.0	
Sex					
Male	37	11.0	9.0	15.0	0.816
Female	16	11.0	6.0	18.0	
Histology					
Epitheloid	41	12.0	9.0	19.0	0.045
Bifasic	2	20.5	15.0	26.0	
MM	4	11.5	9.0	13.0	
Sarcomatoides	6	1.0	0.0	56.0	
Stage					
Early (I/II)	37	12.0	10.0	20.0	**0.049**
Late (III/IV)	16	9.5	2.0	14.0	
Treatment					**0.002**
CHT	32	11.0	10.0	20.0	
Surgery	1	12.0	0.0	0.0	
MMT	8	26.5	15.0	66.0	
BSC	12	6.0	1.0	13.0	
PS					
0–1	46	12.5	11.0	17.0	
2–3	7	1.0	0.0	102.0	
PD-L1					
TPS < 1%	32	11.0	8.787	13.213	0.799
TPS ≥ 1%	21	12.0	3.028	20.972	
TPS < 10%	43	12.0	9.596	14.404	0.464
TPS ≥ 10%	10	5.0	0.000	14.297	
BAP1					
Loss	43	12.0	9.145	14.855	0.541
Positive	10	4.0	0.0	14.847	

Abbreviations: OS, overall survival; MM, malignant mesothelioma histologically nonspecific; MMT, multimodality treatment; CHT, chemotherapy; BSC, best supportive care; PD-L1, programmed cell death ligand 1; TPS, tumor proportion score; BAP1, BRCA1-associated protein-1.

## Data Availability

Data are contained within the article.
